# Alterations of the paired maternal fecal microbiota and neonatal meconium microbiota in newborns from pregnant women with hypertensive disorders

**DOI:** 10.3389/fmicb.2025.1567721

**Published:** 2025-04-16

**Authors:** Heng Yang, Zhijiang He, Jianfen Lai, Jing Yang, Qianrong Huang, Ying Chang, Mingyuan Tian, Hongli Huang

**Affiliations:** ^1^Department of Obstetrics and Gynecology, Shenzhen Luohu Maternity and Child Health Hospital, Shenzhen, China; ^2^Department of Paediatrics, Shenzhen University General Hospital, Shenzhen, China; ^3^Department of Geriatrics, Chongqing General Hospital, Chongqing University, Chongqing, China; ^4^Department of Endocrinology and Metabolism, The Second Affiliated Hospital of Chongqing Medical University, Chongqing, China

**Keywords:** hypertensive disorders of pregnancy, pre-eclampsia, gestational hypertension, maternal fecal microbial community, neonatal meconium microbiota

## Abstract

**Background:**

Hypertensive disorders of pregnancy (HDP) pose significant risks to both maternal and fetal health and have been associated with alterations in the maternal gut microbiota. However, the impact of HDP on neonatal microbiota remains poorly understood. This study aimed to characterize the gut microbiota of pregnant women with HDP and evaluate its potential influence on the meconium microbiota of their newborns.

**Methods:**

A cohort of 67 pregnant women, including 36 diagnosed with HDP (HDP group) and 31 healthy, age-matched controls (HC group), along with their offspring, were recruited. Fecal samples collected during the third trimester and meconium samples from the newborns were subjected to microbial community profiling via 16S rRNA gene sequencing.

**Results:**

Principal coordinate analysis (PCoA) based on Bray-Curtis distances revealed significant differences in microbial community composition between the HDP and HC groups in both maternal and neonatal samples. Subgroup analyses, stratified by HDP severity and medication use, further delineated distinct microbial profiles relative to controls. Notably, both maternal and neonatal microbiota in the HDP group exhibited increased abundances of *Enterobacter*, *Klebsiella*, and *Sphingomonas*, coupled with a reduction in *Acidovorax*, *Azospirillum*, *Caulobacter*, *Flavobacterium*, *Magnetospirillum*, and *Rubrivivax* compared to the HC group. Moreover, the P4-PWY pathway, which is involved in the biosynthesis of L-lysine, L-threonine, and L-methionine, was differentially represented in both maternal and neonatal microbiota in the HDP group. These parallel patterns suggest an intergenerational concordance associated with HDP.

**Conclusion:**

This study demonstrates significant alterations in the microbial communities of both maternal fecal and neonatal meconium samples in the context of HDP. The findings highlight the importance of further research to elucidate the long-term health implications of HDP-associated microbiota shifts on offspring.

## Introduction

Hypertensive disorder of pregnancy (HDP) represent a prevalent and serious medical complication that significantly contributes to maternal morbidity and mortality (Ramos Filho and Antunes, 2020). HDP encompasses a spectrum of conditions, including gestational hypertension, preeclampsia/eclampsia, chronic hypertension, and chronic hypertension complicated with preeclampsia/eclampsia (Brown et al., 2018; Dines and Kattah, 2020). The global incidence of HDP is rising, posing an increasing public health challenge (Garovic et al., 2020). In China, HDP affects approximately 7.3% of all pregnancies (Li et al., 2021). Beyond its immediate consequences, such as preterm delivery, low birth weight, and neonatal care unit admission (Abdelazim et al., 2020), HDP has enduring impacts on both maternal and offspring health. These long-term effects include an increased risk of high blood pressure, coronary heart disease, impaired neonatal brain development, and systemic vascular dysfunction (Garovic et al., 2020; Kanata et al., 2021). Elucidating the underlying mechanisms of HDP and its association with offspring health outcomes is essential for advancing clinical management and developing preventive strategies that benefit both mothers and their children.

Recent investigations have linked HDP with various maternal and placental factors, including inflammatory response, endothelial dysfunction, and alterations in the gut microbiota (Beckers and Sones, 2020; Yu et al., 2022). In particular, dysbiosis in the maternal gut microbiota has been reported in HDP cases, characterized by reduced microbial diversity, diminished short-chain fatty acid-producing bacteria, and an enrichment of potentially pathogenic genera, such as *Enterobacter* and *Klebsiella* (Beckers and Sones, 2020; Chang et al., 2020). This dysbiosis is hypothesized to contribute to the onset and progression of HDP by disrupting maternal physiological homeostasis (Zhang et al., 2015; Liu et al., 2017; Beckers and Sones, 2020).

The initial establishment of the infant gut microbiota, which plays a pivotal role in long-term health, is also influenced by maternal factors. Variables such as antibiotic exposure, delivery mode, and feeding practices significantly shape the neonatal microbial composition (Singh and Mittal, 2020). The maternal microbial reservoir, including the gut, oral, skin, and vaginal microbiota, critically contributes to the infant’s initial microbial colonization (Ferretti et al., 2018). For instance, the association between maternal conditions such as gestational diabetes and alterations in the meconium microbiota is well-documented (Gosalbes et al., 2013; Hu et al., 2013; Wang et al., 2018; Chen et al., 2021; Sililas et al., 2021). Neonates born to mothers with gestational diabetes exhibit reduced alpha diversity and notable shifts in the abundance of Firmicutes and Proteobacteria (Chen et al., 2021).

Despite these advances, the potential impact of HDP on the neonatal gut microbiota remains insufficiently explored. This gap in knowledge limits our understanding of the interplay between maternal HDP and the development of the offspring’s gut microbiota. Based on these considerations, we hypothesize that maternal HDP alters the neonatal meconium microbiota, contributing to microbial dysbiosis with potential long-term health implications for the offspring. Therefore, this study aims to comprehensively characterize the meconium microbiota of neonates born to mothers with HDP and elucidate the relationship between maternal HDP and alterations in the neonatal gut microbiota, thereby providing novel insights into the mechanisms by which HDP may affect offspring health.

## Materials and methods

### Study subjects

The study protocol was approved by the Ethical Committee of Shenzhen Luohu Maternity and Child Health Hospital. Written informed consent was obtained from all participants, and for neonates, consent was provided by their parents or legal guardians. Between April to November 2020, pregnant women in their third trimester who planned to deliver at our hospital and agreed to provide fecal samples were recruited. Exclusion criteria included pregnancy complications (e.g., gestational diabetes, chronic hypertension, intrahepatic cholestasis of pregnancy, premature delivery, or premature rupture of membrane), multiple gestations, antibiotic treatment within 1 month prior to sample collection, a history of smoking or alcohol consumption during pregnancy, or probiotic supplementation. Neonates with significant congenital anomalies, neurological dysfunction, chromosomal abnormalities, or metabolic diseases were also excluded. A total of 67 singleton pregnant women with full-term births were included in the final analysis. Of these, 36 women diagnosed with hypertensive disorders of pregnancy (HDP) were assigned to the HDP group, which comprised 23 with preeclampsia (PE subgroup) and 13 with gestational hypertension (GH subgroup). The remaining 31 normotensive women without complications served as the healthy control (HC) group. Diagnostic criteria for GH included a systolic blood pressure ≥ 140 mmHg and/or a diastolic blood pressure ≥ 90 mmHg, whereas PE was defined as a systolic blood pressure ≥ 140 mmHg and/or a diastolic blood pressure ≥ 90 mmHg in conjunction with proteinuria exceeding > 0.3 g/24 h after 20 weeks of gestation. Clinical information were recorded and verified by trained clinicians following standard procedures.

### Sample collection

Fecal samples from pregnant women were collected during the third trimester, approximately 3 days before labor, using sterile swabs under the guidance of trained nursing staff. Meconium samples from the neonates were obtained within the first hours after birth using sterile swabs by experienced nurses in the labor ward. Approximately 200 mg of meconium was carefully collected directly from the interior of soiled diapers to ensure representative sampling and minimize potential contamination from external components (Chen et al., 2021). All samples were immediately transferred into sterile tubes, stored at −80°C to preserve their integrity, and subsequently processed for further analysis.

### DNA extraction and 16S rRNA sequencing

Genomic DNA was extracted from both fecal and meconium samples using the QIAamp Fast DNA Stool Mini kit, following the manufacturer’s protocol. The V4 hypervariable region of the 16S rRNA gene was amplified by PCR using primers 515F and 806R, which included unique barcodes for sample identification (Kozich et al., 2013). PCR amplification was performed with the Phusion High-Fidelity PCR Master Mix, and the amplified products were pooled in equimolar concentrations and purified using the Qiagen Gel Extraction kit. Sequencing libraries were prepared with the TruSeq DNA PCR-Free Sample Preparation kit, and their quality was assessed using a Qubit@2.0 Fluorometer and Agilent Bioanalyzer 2,100. Libraries were sequenced on an Illumina HiSeq 2,500 platform (V3 chemistry) at Novogene Bioinformatics Technology Co., Ltd., generating 250-bp paired-end reads.

### Bioinformatics and statistical analyses

Raw16S rRNA gene sequences were processed using QIIME2 (Bolyen et al., 2019). Paired-end reads were assembled and denoised with the DADA2 algorithm (Callahan et al., 2016). Taxonomic assignments were performed against the GreenGenes database using the Naïve Bayes classifier in QIIME2. Alpha and beta diversity metrics were calculated using QIIME2’s core diversity tools, and functional metabolic pathways were predicted using PICRUSt2.0 (Douglas et al., 2020).

Microbial community dissimilarities were evaluated through principal coordinate analysis (PCoA) based on Bray-Curtis distances, and statistical significance was assessed using permutational multivariate analysis of variance (PERMANOVA) implemented in the R package MicrobiotaProcess (Xu et al., 2023). Differentially abundant taxa were identified using LEfSe, with a linear discriminant analysis (LDA) score > 2.0 and *p* < 0.05 (Segata et al., 2011). Pathway differences between the HDP and HC groups were analyzed using STAMP with Bonferroni correction (Parks et al., 2014).

Clinical characteristics were summarized as means and standard deviations for continuous variables and proportions for categorical variables. Between-group differences were assessed using *t*-tests for continuous variables and chi-square tests for categorical variables. All statistical analyses were performed using R software (version 3.6.3), with significance set at *p* < 0.05.

## Results

### Characteristics of study participants

**Figure 1:**
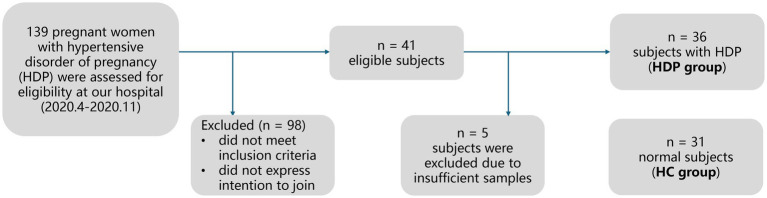
Flowchart depicting the study population and design.

Figure 1 illustrates the recruitment and exclusion process. A total of 67 full-term neonate-mother pairs were included, comprising 36 pairs in the HDP group, and 31 pairs in the HC group. All participants were Han Chinese residing in Shenzhen, China. Table 1 summarizes the sociodemographic and clinical characteristics. There were no significant differences between the HDP and HC groups in terms of gestational age, birth weight, birth length, neonatal gender, maternal age, or maternal pre-pregnancy BMI. Among the mothers in the HDP group, 14 received labetalol, two were treated with nicardipine, and one received nifedipine during pregnancy.

**Table 1:** Characteristics of the newborns of mothers with and without HDP.

	HDP (*n* = 36)	HC (*n* = 31)	*p*-value
Maternal characteristics			
Age at delivery, years	31.2 ± 5.0	29.7 ± 5.1	0.23
Pre-pregnancy BMI (kg/m2)	23.2 ± 4.1	21.6 ± 2.5	0.07
Medical treatment	17	0	<0.01*
Neonatal characteristics			
Gestational age, weeks	38.9 ± 2.2	39.5 ± 0.9	0.14
Apgar score	10	10	1
Birth weight, g	3,250 ± 434	3,327 ± 326	0.43
Birth length, cm	50 ± 1.2	50.2 ± 0.8	0.55
Gender (male/female)	20/16	18/11	0.78

*Means significantly different.

### Comparison of neonatal meconium microbiota between the HDP and HC groups

Sequencing of neonatal meconium samples yielded a total of 6,634,295 paired end reads, with an average of 96,128 per sample for the HDP group and 102,376 reads per sample for the HC group. And a total of 16,190 amplicon sequence variants were identified. Rarefaction analysis indicated that all samples reached an asymptote, suggesting that sufficient sequencing depth was achieved (data not shown).

**Figure 2:**
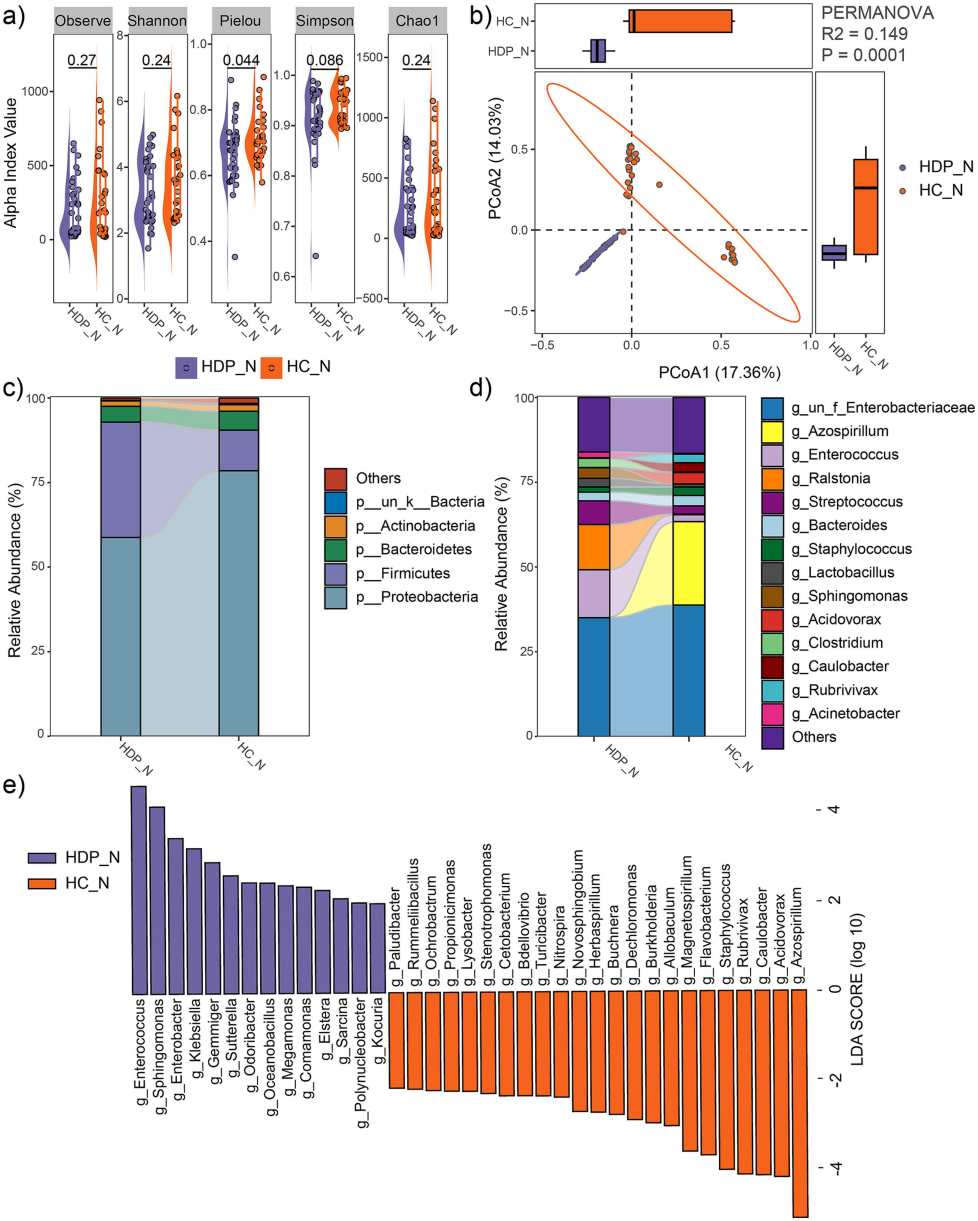
Comparison of neonatal meconium microbial communities between the HDP (HDP_N) and HC (HC_N) groups. **(a)** Comparisons of alpha diversity indices using two-tailed Student’s *t*-test. **(b)** PCoA plot illustrating differences in microbial community composition between groups, as assessed by PERMANOVA. **(c)** Relative abundances of the dominant phyla. **(d)** Relative abundances of the predominant genera. **(e)** Genera with significantly different relative abundances between the HDP_N and HC_N groups, identified using LEfSe software with an LDA score > 2.0 and *p* < 0.05.

Alpha diversity indices, including observed feature number, Pielou index, Chao1 index, Simpson index and Shannon index, were compared between the HDP and HC groups. While no significant differences were observed in the observed feature number, Chao1, Simpson and Shannon indices, the Pielou index was significantly lower in the HDP group compared to the HC group (*p* = 0.044; Figure 2a). Moreover, principal coordinate analysis based on Bray-Curtis distances revealed a distinct separation between the HDP and HC groups (R^2^ = 0.149, *p* = 0.0001; Figure 2b).

At the phylum level, the predominant taxa in the neonatal meconium were Proteobacteria, Firmicutes, Bacteroidetes, and Actinobacteria (Figure 2c), with Firmicutes being significantly more abundant in the HDP group (LDA = 4.96, *p* = 0.001). Notably, the combined relative abundances of Firmicutes and Proteobacteria exceeded 90% in both groups. At the genus level, the dominant taxa included *Azospirillum*, *Enterococcus*, *Ralstonia*, *Streptococcus*, *Bacteroides*, *Staphylococcus*, *Lactobacillus*, *Sphingomonas*, *Acidovorax*, *Clostridium*, *Caulobacter*, *Rubrivivax*, and *Acinetobacter* (Figure 2d). LEfSe analysis revealed significant differences in microbial composition between the groups. The HDP group showed higher relative abundances of *Enterococcus*, *Sphingomonas*, *Enterobacter*, *Klebsiella*, *Gemmiger*, *Sutterella*, *Odoribacter*, *Megamonas*, *Comamonas*, *Elstera*, *Sarcina*, *Polynucleobacter*, and *Kocuria*. In contrast, lower relative abundances in the HDP group were observed for *Azospirillum*, *Acidovorax, Caulobacter, Rubrivivax*, *Staphylococcus*, *Flavobacterium*, *Magnetospirillum*, *Allobaculum*, *Burkholderia*, *Dechloromonas*, *Buchnera*, *Herbaspirillum*, *Novosphingobium*, *Nitrospira*, *Turicibacter*, *Bdellovibrio*, *Cetobacterium*, *Stenotrophomonas*, *Lysobacter*, *Propionicimonas*, *Ochrobactrum*, *Rummeliibacillus*, and *Paludibacter* (Figure 2e).

### Comparison of maternal fecal microbial community between the HDP and HC groups

Sequencing of maternal fecal samples generated a total of 6,135,597 paired end reads, with an average of 101,412 reads per sample in the HDP group and 80,153 reads per sample in the HC group. A total of 12,839 amplicon sequence variants were identified and rarefaction analysis confirmed that all samples reached an asymptote, indicating sufficient sequencing depth (data not shown).

**Figure 3:**
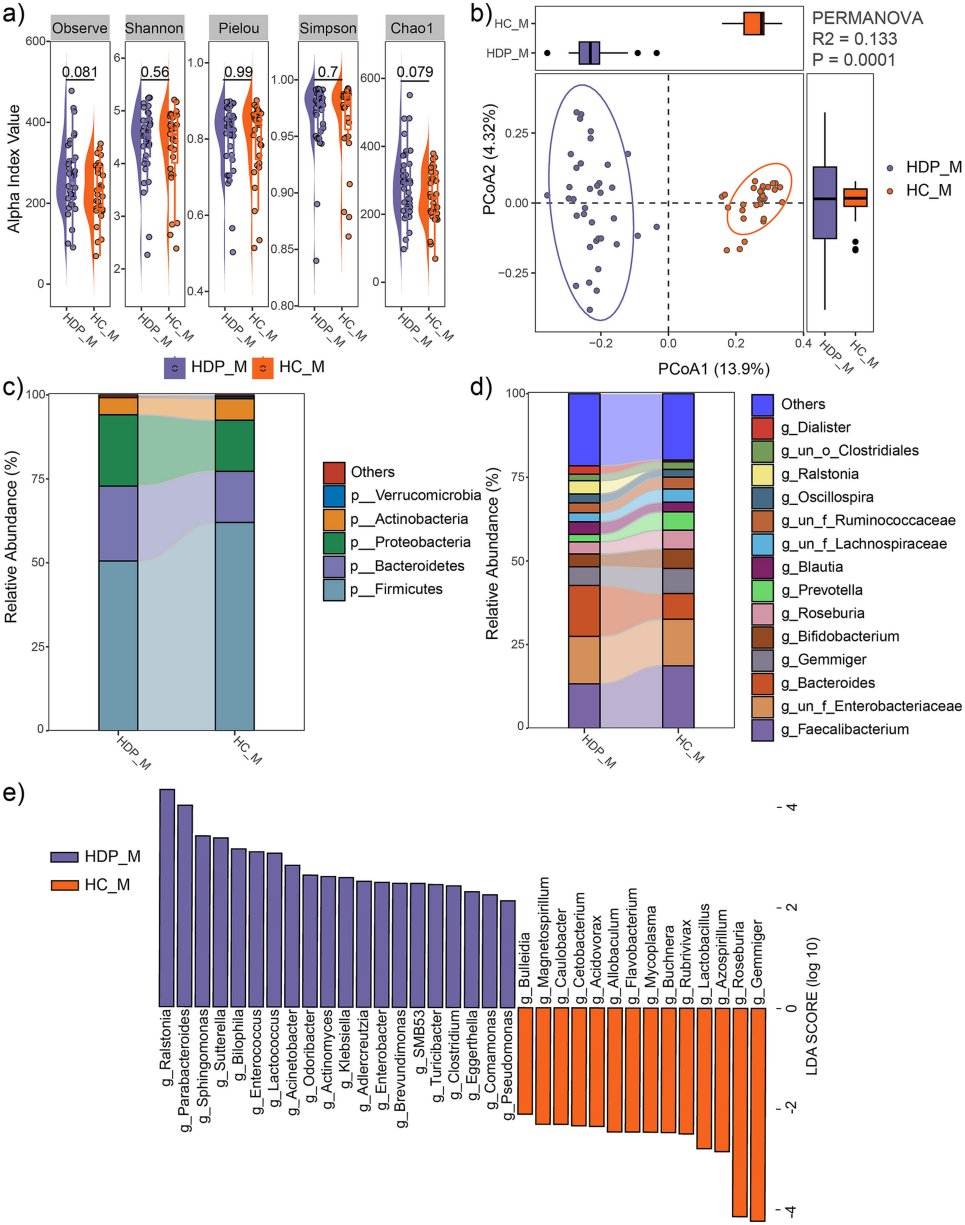
Comparison of maternal fecal microbial communities between the HDP (HDP_M) and HC (HDP_M) groups. **(a)** Comparisons of alpha diversity indices using *t*-tests. **(b)** PCoA plot illustrating the differences in microbial community composition between the two groups, as determined by PERMANOVA. **(c)** Relative abundances of the dominant phyla. **(d)** Relative abundances of the predominant genera. **(e)** Genera with significantly different relative abundances between the HDP_M and HC_M groups, as determined by LEfSe (LDA score > 2.0 and *p* < 0.05).

No significant differences in alpha diversity metrics were observed between the HDP and HC groups (Figure 3a). However, PCoA based on Bray-Curtis distances demonstrated a significant separation between the two groups (R^2^ = 0.133, *p* = 0.0001; Figure 3b).

At the phylum level, the maternal fecal microbiota was predominantly composed of Firmicutes, Bacteroidetes, Proteobacteria, and Actinobacteria (Figure 3c), with Proteobacteria being significantly more abundant in the HDP group (LDA = 4.65, *p* = 0.0064). At the genus level, dominant taxa included *Faecalibacterium*, *Bacteroides*, *Gemmiger*, *Bifidobacterium*, *Roseburia*, *Prevotella*, *Blautia*, *Oscillospira*, *Ralstonia*, and *Dialister* (Figure 3d). LEfSe analysis further revealed significant differences in microbial composition between the groups. In the HDP group, genera such as *Ralstonia*, *Parabacteroides*, *Sphingomonas*, *Sutterella*, *Bilophila*, *Enterococcus*, *Lactococcus*, *Acinetobacter*, *Odoribacter*, *Actinomyces*, *Klebsiella*, *Adlercreutzia*, *Enterobacter*, *Brevundimonas*, *SMB53*, *Turicibacter*, *Clostridium*, *Eggerthella*, *Comamonas*, and *Pseudomonas* were significantly enriched, whereas *Bulleidia*, *Magnetospirillum, Caulobacter, Cetobacterium*, *Acidovorax, Allobaculum*, *Flavobacterium*, *Mycoplasma*, *Buchnera*, *Rubrivivax*, *Lactobacillus*, *Azospirillum*, *Roseburia*, and *Gemmiger* were significantly depleted (Figure 3e).

Notably, an intergenerational concordance was evident, as genera such as *Enterococcus*, *Sphingomonas*, *Enterobacter*, *Klebsiella*, *Sutterella*, *Odoribacter*, and *Comamonas* were significantly increased in both the maternal fecal and neonatal meconium microbiota of the HDP group, while *Azospirillum*, *Acidovorax, Caulobacter, Rubrivivax*, *Flavobacterium*, *Magnetospirillum*, *Allobaculum*, *Buchnera*, *Turicibacter*, and *Cetobacterium* were significantly decreased in both compartments.

### Comparison of functionally predicted metabolic pathways between the HDP and HC groups

**Figure 4:**
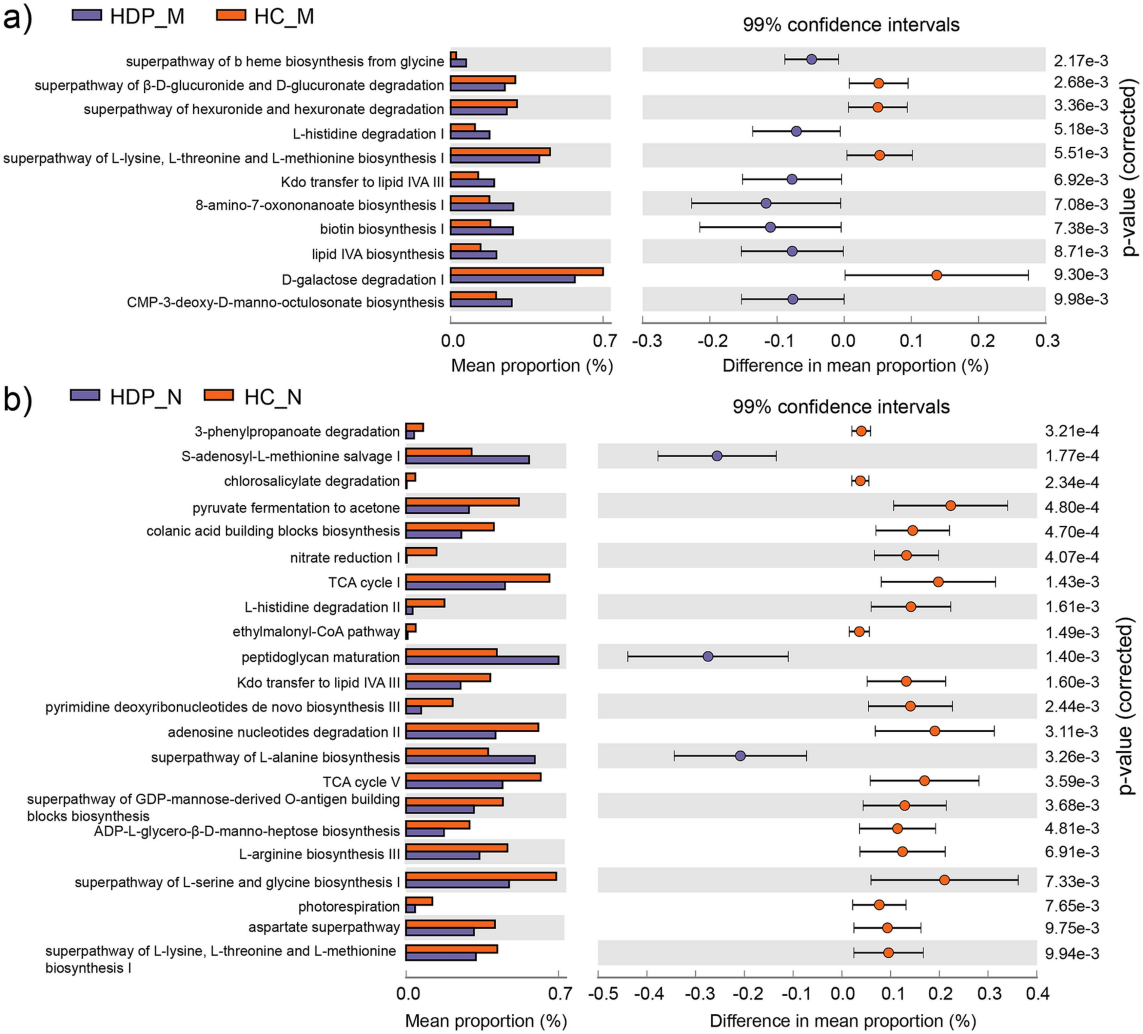
Differentially abundant metabolic pathways predicted by PICRUSt2 between the HDP and HC groups. **(a)** Differential metabolic pathways identified in the maternal gut microbiota between the HDP_M and HC_M groups with adjusted *p* value less than 0.01. **(b)** Differential metabolic pathways identified in the neonatal meconium microbiota between the HDP_N and HC_N groups with adjusted p value less than 0.01.

Functional annotation was performed using PICRUSt2.0 against the MetaCyc database to assess the metabolic potential of the maternal fecal and neonatal meconium microbiota associated with HDP. In maternal fecal samples, 11 metabolic pathways exhibited significant differences between the HDP and HC groups (Figure 4a), while 22 metabolic pathways were significantly altered in the neonatal meconium microbiota (Figure 4b). Notably, the “superpathway of L-lysine, L-threonine and L-methionine biosynthesis I (P4-PWY)” and “Kdo transfer to lipid IV_A_ III (Chlamydia; PWY-6467)” pathways were significantly altered in both maternal and neonatal samples, suggesting shared functional impairments linked to HDP.

### Comparison of microbial community between the sub-HDP groups and HC group

**Figure 5:**
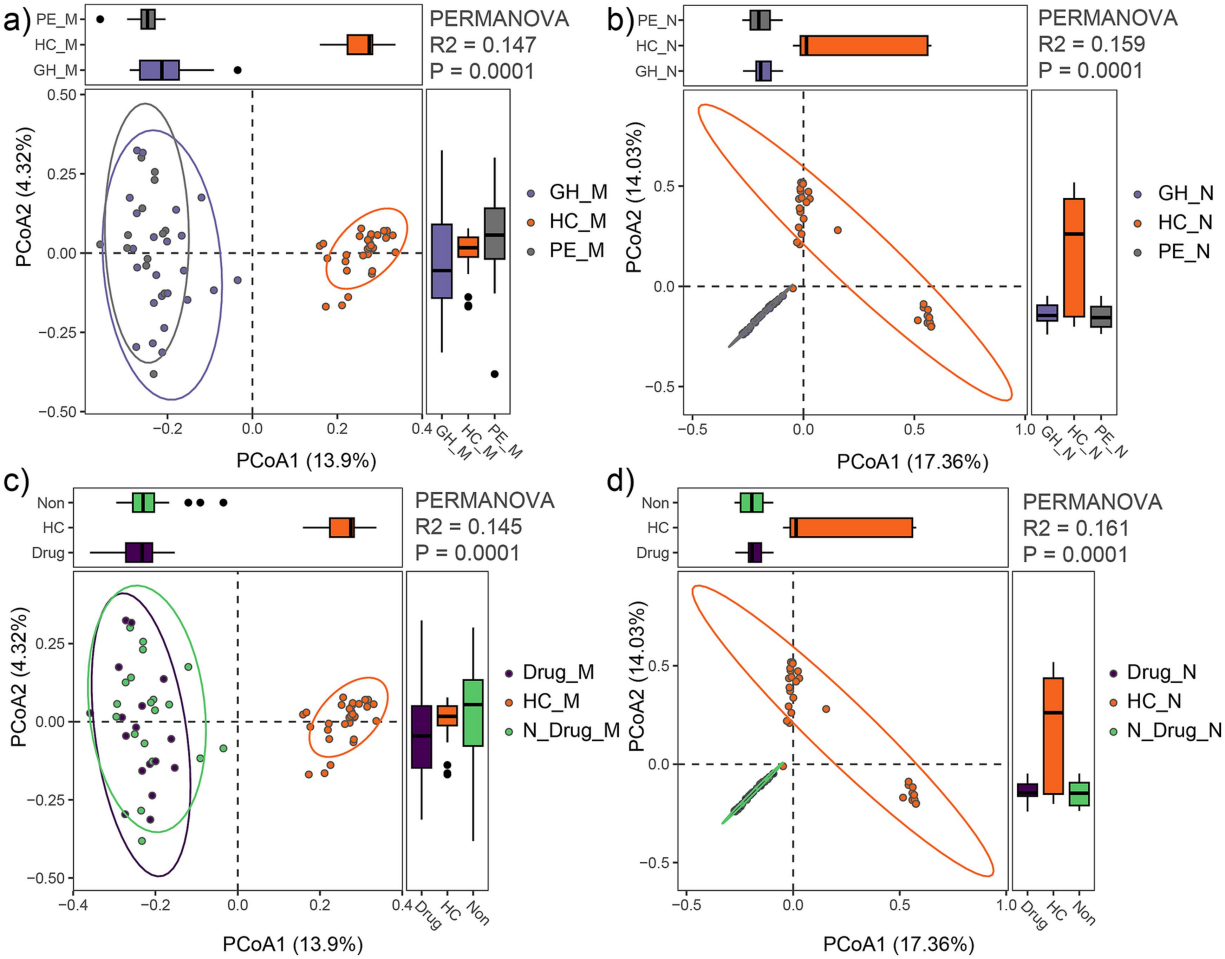
Comparison of gut microbial communities among HDP subgroups (GH, PE) and HC group. **(a)** PCoA plot showing differences in maternal fecal microbial communities among the GH_M, PE_M and HC_M groups. **(b)** PCoA plot showing differences in neonatal meconium microbial communities among the GH_N, PE_N and HC_N groups. **(c)** PCoA plot illustrating differences in maternal fecal microbial communities among mothers receiving drug treatment (Drug_M), those not receiving drug treatment (N_Drug_M), and HC (HC_M) groups. **(d)** PCoA plot illustrating differences in neonatal meconium microbial communities among neonates born to mothers receiving drug treatment (Drug_N), those not receiving drug treatment (N_Drug_N), and HC (HC_N) groups.

Based on clinical data, the HDP group was subdivided into the gestational hypertension (GH, *n* = 13) and preeclampsia (PE, *n* = 23) subgroups. PCoA with PERMANOVA revealed that the maternal fecal microbial community of the PE and GH subgroups were clustered closely (R^2^ = 0.03, *p* = 0.54), as did their neonatal meconium microbiota (R^2^ = 0.02, *p* = 0.85). However, when comparing the PE, GH, and HC groups, significant differences in microbial composition were observed. The maternal fecal microbial community showed marked separation among these groups (R^2^ = 0.147, *p* = 0.0001; Figure 5a), with similar findings in the neonatal meconium microbiota (R^2^ = 0.159, *p* = 0.0001; Figure 5b). Furthermore, subgroup analysis based on medication use confirmed significant variation between the HDP and HC groups for both maternal microbiota (R^2^ = 0.145, *p* = 0.0001; Figure 5c) and neonatal microbiota (R^2^ = 0.161, *p* = 0.0001; Figure 5d). Notably, within the HDP group, no significant differences were observed in microbial composition between mothers treated with antihypertensive drugs (labetalol, nicardipine, or nifedipine) and those who were untreated (R^2^ = 0.022, *p* = 0.646). These findings underscore the distinct microbial profiles associated with HDP compared to healthy controls.

## Discussion

In this study, we investigated the neonatal meconium microbiota in relation to the maternal fecal microbial community among mothers with HDP using high throughput 16S rRNA sequencing. Our analysis revealed that the meconium microbiota was predominantly composed of the phyla Proteobacteria and Firmicutes, consistent with previous reports (Bäckhed et al., 2015; Turunen et al., 2021). In contrast, the maternal fecal microbial community was mainly comprised of Firmicutes and Bacteroidetes, in agreement with earlier findings (Yu et al., 2022).

Principal coordinate analysis based on Bray-Curtis distances demonstrated a significant separation between the HDP and HC groups in the neonatal meconium microbiota. Moreover, we observed marked alterations in the maternal fecal microbial community of women with HDP, which corroborates previous studies (Gomez-Arango et al., 2016; Chang et al., 2020). These differences persisted across subgroups stratified by disease severity and medication use during pregnancy. Specifically, genera such as *Klebsiella*, *Enterobacter*, and *Sphingomonas* were significantly enriched in both the maternal and neonatal microbiota of the HDP group, whereas *Acidovorax*, *Azospirillum*, *Caulobacter*, *Flavobacterium*, *Magnetospirillum*, and *Rubrivivax* were more abundant in the HC group. Notably, previous research has associated certain species of *Klebsiella* (You et al., 2020; Mukherjee et al., 2023)*, Enterobacter* (Ferry et al., 2020; Karambin and Zarkesh, 2011), and *Sphingomonas* (Mutlu et al., 2011; Bayram et al., 2013; Ranjan et al., 2016) with neonatal infection. Furthermore, a decrease in *Azospirillum* has been linked to helminth infections in preschool-aged children (Osakunor et al., 2020), while genera such as *Acidovorax*, *Caulobacter*, *Flavobacterium*, and *Magnetospirillum* have been detected in the gut microbiota (Simon et al., 2021; Dornelles et al., 2022; Xu et al., 2022). Increased abundance of *Enterobacter* and *Klebsiella* have also been reported in individuals with PE (Beckers and Sones, 2020; Chang et al., 2020). Both genera are known to secrete lipopolysaccharides, potent activators of the immune system that can elicit strong inflammatory responses (Holt et al., 2020). Such immune activation may contribute to an elevated risk of preterm labor and other complications. Although these microbial alterations may adversely affect neonatal health and development, the specific roles of these taxa in neonates born to mothers with HDP require further investigation.

Additionally, previous work by de Agüero MG et al. identified a high abundance of *Lactobacillus* spp. in the placental microbiota of healthy term deliveries (Gomez De Agüero et al., 2016), suggesting a beneficial role for *Lactobacillus* in pregnancy outcomes (Olaniyi et al., 2020). Consistent with these observations, our study found a significantly lower level of *Lactobacillus* in the maternal fecal microbial community of the HDP group compared to controls. Furthermore, *Roseburia*, an abundant genera associated with several diseases such as obesity, type 2 diabetes, nervous system conditions and allergies (Tamanai-Shacoori et al., 2017), was significantly reduced in the HDP group. *Roseburia* is a key producer of short-chain fatty acids, particularly butyrate, which is crucial for maintaining colonic motility, immunity function and anti-inflammatory responses (Tamanai-Shacoori et al., 2017; Yan et al., 2017; Calderón-Pérez et al., 2020). These findings suggest that reduced colonization by lactic acid and short-chain fatty acids producing bacteria may contribute to adverse pregnancy outcomes. However, larger prospective studies are needed to confirm this hypothesis.

Moreover, functional prediction analysis revealed that the abundance of the P4-PWY (superpathway of L-lysine, L-threonine and L-methionine biosynthesis I) was significantly lower in both the neonatal and maternal microbiota of the HDP group compared to the HC group. Considering the essential roles of L-lysine in protein synthesis and neonatal growth (van der Schoor et al., 2004), as well as the importance of maternal methionine supply for offspring development (Alharthi et al., 2018; Elolimy et al., 2019), these alterations in key metabolic pathways may have significant implications for neonatal health.

Despite these important insights, several limitations warrant consideration. The relatively small sample size and recruitment from a single geographic area may limit the generalizability of the findings. Additionally, the absence of long-term clinical follow-up data restricts our ability to assess the direct impact of gut microbial alterations on neonatal health outcomes. Future studies employing metagenomic sequencing, longitudinal follow-up, and animal models are necessary to elucidate the mechanistic links between HDP-related microbial dysbiosis and neonatal development. Such efforts will enhance the robustness and applicability of these findings and pave the way for more insightful research in this critical area.

In conclusion, our study demonstrates significant alterations in both the neonatal meconium microbiota and maternal fecal microbial community in the context of hypertensive disorders of pregnancy. These findings advance our understanding of the impact of HDP on neonatal gut microbiota. Further research is necessary to explore the potential implications of these microbial alterations on neonatal health outcomes and to develop strategies for modulating the gut microbiota in this vulnerable population.

## Data Availability

The datasets presented in this study can be found in online repositories. The names of the repository/repositories and accession number(s) can be found at: https://db.cngb.org/search/project/CNP0003728/, CNP0003728.
